# Habitual coffee drinkers display a distinct pattern of brain functional connectivity

**DOI:** 10.1038/s41380-021-01075-4

**Published:** 2021-04-20

**Authors:** Ricardo Magalhães, Maria Picó-Pérez, Madalena Esteves, Rita Vieira, Teresa C. Castanho, Liliana Amorim, Mafalda Sousa, Ana Coelho, Henrique M. Fernandes, Joana Cabral, Pedro S. Moreira, Nuno Sousa

**Affiliations:** 1 Life and Health Sciences Research Institute (ICVS), School of Medicine, University of Minho, Campus de Gualtar, Braga, Portugal; 2 ICVS/3B’s, PT Government Associate Laboratory, Braga/Guimarães, Portugal; 3 Clinical Academic Center - Braga, Braga, Portugal; 4 NeuroSpin, CEA, CNRS, Paris-Saclay University, Gif-sur-Yvette, France; 5 Center for Music in the Brain, Department of Clinical Medicine, Aarhus University, Aarhus, Denmark; 6 Psychological Neuroscience Lab, CIPsi, School of Psychology, University of Minho, Braga, Portugal; 7 P5 Medical Center, Braga, Portugal

**Keywords:** Neuroscience, Biomarkers

## Abstract

Coffee is the most widely consumed source of caffeine worldwide, partly due to the psychoactive effects of this methylxanthine. Interestingly, the effects of its chronic consumption on the brain’s intrinsic functional networks are still largely unknown. This study provides the first extended characterization of the effects of chronic coffee consumption on human brain networks. Subjects were recruited and divided into two groups: habitual coffee drinkers (CD) and non-coffee drinkers (NCD). Resting-state functional magnetic resonance imaging (fMRI) was acquired in these volunteers who were also assessed regarding stress, anxiety, and depression scores. In the neuroimaging evaluation, the CD group showed decreased functional connectivity in the somatosensory and limbic networks during resting state as assessed with independent component analysis. The CD group also showed decreased functional connectivity in a network comprising subcortical and posterior brain regions associated with somatosensory, motor, and emotional processing as assessed with network-based statistics; moreover, CD displayed longer lifetime of a functional network involving subcortical regions, the visual network and the cerebellum. Importantly, all these differences were dependent on the frequency of caffeine consumption, and were reproduced after NCD drank coffee. CD showed higher stress levels than NCD, and although no other group effects were observed in this psychological assessment, increased frequency of caffeine consumption was also associated with increased anxiety in males. In conclusion, higher consumption of coffee and caffeinated products has an impact in brain functional connectivity at rest with implications in emotionality, alertness, and readiness to action.

## Introduction

Coffee is the most widely consumed beverage, with particular interest for human health in view of its short-term effects on attention, sleep, and memory and its long-term impact on the appearance of different diseases and on healthy span of ageing [1, 2]. Coffee has several constituents able to impact on human health, amongst which stems caffeine, which is the most widely consumed psychostimulant in the world [3]. Despite its widespread use it is surprising to note that a thorough characterization of the chronic effects of coffee upon the human brain is still lacking. In the present work we aim to begin addressing that issue.

In the brain, caffeine acts as an antagonist of adenosine A1 and A2A receptors, leading to hyperexcitability of the central nervous system [3, 4]. This induces acute effects in diverse domains, such as physical endurance [1, 5], vigilance, dexterity [6], mood [7, 8], memory [9], and cognitive function [1, 8, 10]. There is also evidence that coffee/caffeine intake can normalize anxiety [11], although higher doses of caffeine may be anxiogenic [1, 12] by disrupting the HPA axis [13]. On the other hand, epidemiological and animal studies converge in concluding that coffee, caffeine and adenosine receptor antagonists attenuate the burden of neurodegenerative disorders such as Alzheimer’s [14], or psychiatric disorders such as depression [15]. Indeed, chronic antagonism of either A1 or A2 receptors seems to induce an upregulation of the former, but not the latter. The resulting altered receptor ratio may explain the shift from the acute psychomotor effects (e.g., attention, vigilance) to the longer-term actions of coffee (e.g., stress resistance, neuroprotection) effects [4, 16].

Functional magnetic resonance imaging (fMRI) allows studying, in a noninvasive way, the function of the human brain during execution of different tasks or at rest [17]. So far, most studies using fMRI were focused on measuring the acute effects of caffeine intake in the brain. Briefly, they have reported caffeine-related increases in blood oxygenation-dependent-level (BOLD) signal in different cortical and subcortical areas during a visuomotor task [18]; an impact in working memory and perfusion in elderly subjects [19, 20]; an increase in BOLD activation in the frontopolar and cingulate cortex during a 2-back verbal working memory task [21, 22]; and a global caffeine-induced increase in brain entropy, possibly representing an increased processing capacity [23]. Very few studies, however, were performed to study the acute effects of caffeine in functional connectivity (FC) at rest [24, 25]. Those few studies reveal a general trend for a caffeine-induced reduction in FC, associated with neuro-electric power fluctuations as measured through magnetoencephalography and exacerbated anticorrelations. Despite this existing literature, many aspects of the characterization of the impact of caffeine on the brain remain unexplored. Critical amongst these is the characterization of the chronic effects of habitual coffee and caffeine consumption upon the functional architecture of the brain. We are only aware of a single study that touched on this subject [26]. That work revealed an association between different habits of coffee consumption and the magnitude of BOLD signals in the visual cortex; however, it did not address possible effects on the functional connectome or resting state networks. Pursuit of the latter can present significant challenges in finding and recruiting participants with sufficient variation in consumption habits and who are willing to undergo necessary, even if short, abstinence procedures.

To tackle this gap, herein we will use whole brain approaches [27–29], as well as the study of brain functional dynamics [30] to compare FC and its dynamics between habitual and non-habitual coffee consumers. In addition, and because of the potential anxiogenic and HPA-disrupting role of caffeine, measures of psychological state (depression, anxiety, and stress) will also be acquired, in order to explore the potential association of habitual coffee consumption with these variables.

## Methods

### Subject recruitment and assessment

Participants were recruited through advertisement on the Institute’s social media, institutional e-mail, and press releases distributed among Portuguese local and national newspapers. Exclusion criteria included the presence of neurological or psychiatric disorders, habitual consumption of mind-altering substances, and the inability to undergo MRI. Two experimental groups were created according to participants’ coffee consumption habits: coffee drinkers (CD), who drank a minimum of one cup of caffeinated coffee per day; and non-coffee drinkers (NCD), who had no habits of regular consumption of coffee (less than one cup per week). Consumption of coffee as well as other caffeinated products was confirmed in a structured interview. Participants were instructed to abstain from caffeinated products for 3 h before the assessment, in order to avoid acute influence of caffeine. Fifty-six subjects were recruited (32 CD and 24 NCD). One participant from the CD group was excluded due to imaging artifacts, rendering a final sample of 31 CD and 24 NCD. Characterization of subjects was done in two (CD) or three (NCD) parts within a 3 h time-period: participants were first interviewed by a certified psychologist. This was followed by an MRI scanning session, and, in the case of the NCD, the first scanning session was followed by ingestion of coffee (Nespresso^®^ Ristretto, ~50 cc) before a rs-fMRI scan ~30 min thereafter. During the interview, the following data were gathered: demographic data; habits of coffee and other caffeinated products consumption; and assessment of depression, anxiety, and stress scores through the Depression, Anxiety and Stress Scales (DASS-21, [31, 32]).

### Demographic and psychological data analysis

CD and NCD groups were compared in terms of sociodemographic variables, frequency of consumption of caffeinated products, and psychological variables. Since the variables did not follow a normal distribution, nonparametric tests were applied (Wilcoxon test). Moreover, multiple regression analyses were performed, aiming to determine the association between daily consumption of caffeinated products such as coffee, tea, chocolate, etc. (0 = <1/day; 1 = 1/day; 2 = 2/day; 3 = 3 or more/day) and the psychological data measured with the DASS-21 questionnaire (controlled for sex, age, and education), independently of the groups. These analyses were performed on Matlab2020a software (The Mathworks, Inc.) and *p* < 0.05 was considered the threshold for statistical significance. Linear regression representations were generated in Prism 7 software (GraphPad Software, Inc.).

### MRI brain imaging

Magnetic resonance imaging scans were conducted using a Siemens Verio 3T (Siemens, Erlangen, Germany) located in Hospital de Braga (Braga, Portugal) using a 32-channel head antenna. The scanning session included as an anatomical acquisition a T1-weighted sagittal magnetization-prepared rapid acquisition with gradient echo (TE/TR = 2420/4.12 ms, FA = 9°, 1 mm^3^ isometric voxel size, Field-of-View = 176 × 256 × 256 mm^3^). The resting-state fMRI (rs-fMRI) acquisition used a multi-band echo planar imaging sequence, CMRR EPI 2D (R2016A, Center for Magnetic Resonance Research, University of Minnesota, Minnesota, USA [33–35]) sensitive to fluctuations in the BOLD contrast (TR/TE = 1000/27 ms, FA = 62°, 2 mm^3^ isometric voxel size, 64 axial slices over an in plane matrix of 100 × 100). The rs-fMRI acquisition had a duration of 7.5 min, during which subjects were instructed to remain with their eyes closed, relaxed, and let their minds wander freely.

### Preprocessing of MRI data

MRI results included in this manuscript were preprocessed using fMRIPrep 1.4.1 ([36]; RRID:SCR_016216), which is based on Nipype 1.2.0 ([37, 38]; RRID:SCR_002502). A full description of the preprocessing pipeline can be found in the Supplementary material.

### Resting-state analysis

#### Independent component analysis

Resting-state network (RSN) maps were analyzed voxel-wise through a probabilistic independent component analysis (ICA) as implemented in Multivariate Exploratory Linear Optimized Decomposition into Independent Components, distributed with FSL [39]. For further details check the Supplementary material.

The RSNs FC was compared between CD and NCD groups, using a nonparametric permutation procedure implemented in the randomize tool from FSL [40]. Threshold-free cluster enhancement (TFCE) was used to detect widespread significant differences and control the family-wise error rate (FWE-R) at *α* = 0.05. In total, 5000 permutations were performed.

#### Static functional connectomics analysis

To assess differences between the two groups in the functional connectome, the mean time series of the 268 regions of the Shen Atlas [41] were extracted. The Pearson correlation between time series, followed by Fisher r-to-Z transformation, were calculated to obtain matrices of FC for each subject. To overcome the issue of multiple comparisons induced by the large number of connections in the network, we applied the network-based statistics (NBS) approach [42]. A total of 5000 permutations were used, together with a FWE corrected network significance of 0.05. For more details check the Supplementary material.

#### Dynamic functional analysis

We applied the leading eigenvector dynamics analysis (LEiDA, [30]) approach to study the changes in the functional dynamics associated with habitual caffeine consumption. Instantaneous FC was calculated for each subject at each time point for all 268 regions of interest of the Shen atlas, using the time series extracted for the static analysis. To help visually identify phase locked (PL) states, the overlap between each anatomical region of each state to the 7 Yeo RSN’s [43], plus two other labels for the cerebellum and subcortical units, was calculated and anatomical units color coded in accordance to the best match. For more details check the reference paper or the Supplementary material.

#### Effects of acute coffee consumption and frequency of caffeine consumption

The significant findings obtained with ICA, NBS, and LEiDA were further explored, aiming to assess the effects of acute coffee consumption in NCD and of frequency of consumption of caffeinated products in both groups. The first were assessed by comparing NCD after coffee consumption with data from CD (independent sample *t*-test) and NCD (before coffee consumption; paired sample *t*-test). The second were evaluated by performing multiple regression analyses following the same approach described for the DASS-21 questionnaire.

## Results

### Demographic analysis

CD and NCD groups did not differ in terms of age (range 19–57; *p* = 0.28; *Z* = 1.09; *r* = 0.15) or number of formal years of education (range 12–25; *p* = 0.07; *Z* = 1.84; *r* = 0.25). Frequency of consumption of caffeinated products was, as expected, higher in the CD group (*p* < 0.001; *Z* = 6.17; *r* = 0.83). Sex distribution was not significantly different between groups (*χ*^2^(1, *N* = 55) = 0.52, *p* = 0.42), despite the CD group presenting a slightly higher proportion of males (41.94%) in comparison with the NCD group (33.33%). Descriptive statistics can be found in Table 1.

**Table 1:** Descriptive statistics of demographic data.

	Coffee drinkers (*n* = 31; 42% male)		Non-coffee drinkers (*n* = 24; 33% male)		Statistical analysis		
	Med	IQR	Med	IQR	*p*	*Z*	*r*
Age	28.0	16.5	29.5	12.5	0.28	1.09	0.15
Education	17.0	2.0	16.0	4.0	0.07	1.84	0.25
Frequency of consumption	3.0	1.0	0.0	0.0	<0.001*	6.17	0.83

Median (Med) and interquartile range (IQR) of age, years of formal education, and frequency of caffeinated drinks’ consumption for coffee drinkers and non-coffee drinkers.

*Statistically significant between-group differences at *p* < 0.05.

### Effect of habitual caffeine consumption on rs-fMRI data

#### Independent components analysis

**Fig. 1:**
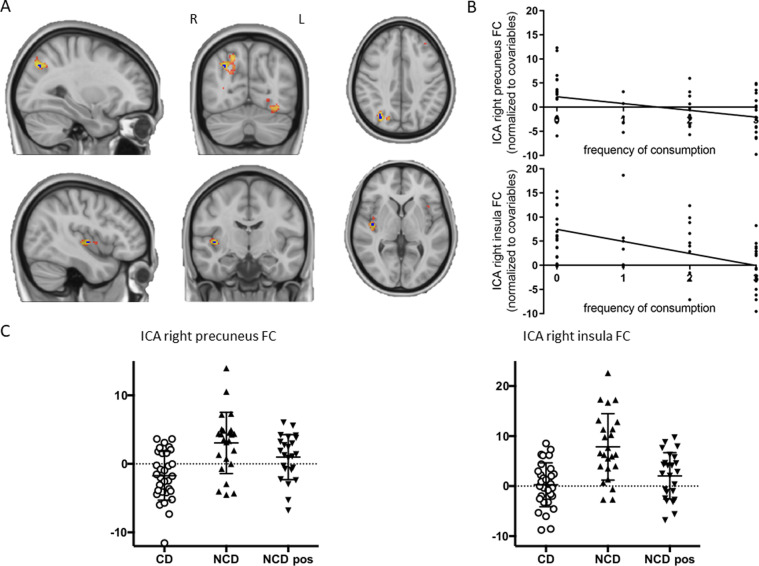
Results from ICA. **A** Sagittal, coronal, and axial view of the clusters showing significant between-group differences in the connectivity between the somatosensory network and the right precuneus (top) and the limbic network and the right insula (bottom). The FWE-R TFCE corrected clusters are shown in dark blue overlaid over a more extended non-significant after multiple comparison correction cluster in hot color scale scheme, for visualization purposes. **B** Associations of frequency of consumption of caffeinated beverages with the mean FC of the right precuneus and the right insula. **C** Scatter plots showing the mean FC of the right precuneus and the right insula for the NCD before drinking coffee (NCD), the NCD after drinking coffee (NCD pos), and the CD.

Thirty components were obtained from the probabilistic ICA of CD and NCD (before consuming coffee). Fifteen of these components were found to be representative of the most typical RSNs. A tendency toward lower FC patterns in the CD group can be seen in most of these networks (see Supplementary Fig. 1). Despite this, we only found significant FWE-R TFCE corrected between-group differences in two of them, namely, in the somatosensory network and the limbic network (Fig. 1). Regarding the somatosensory network, NCD presented a pattern of higher connectivity with the right precuneus (MNI coordinates = 30, −72, 38; 7 voxels; peak *t* value = 4.4). Moreover, for the limbic network, NCD had higher FC in the right insula compared to CD (MNI coordinates = 42, −12, 2; 4 voxels; peak *t* value = 5.09). Of note, these effects were also linearly associated with the caffeinated products’ frequency of consumption. Negative correlations were found for both right precuneus (*p* = 0.003; *β* = −1.433; adjusted *R*^2^ = 0.162; Fig. 1B) and right insula (*p* < 0.001; *β* = −2.384; adjusted *R*^2^ = 0.267; Fig. 1B). Detailed statistics can be found in Supplementary Table 1.

Importantly, the group differences described were reduced after NCD drank coffee (see Fig. 1C; somatosensory network: pre vs post NCD *t* value = 1.86, *p* = 0.075, post NCD vs CD *t* value = −2.89, *p* = 0.006; limbic network: pre vs post NCD *t* value = 3.88, *p* < 0.001, post NCD vs CD *t* value = −1.46, *p* = 0.15). This points to a potential causality link between coffee drinking and the above-described changes in lower connectivity in the somatosensory and in the limbic networks.

#### Connectomics analysis

**Fig. 2:**
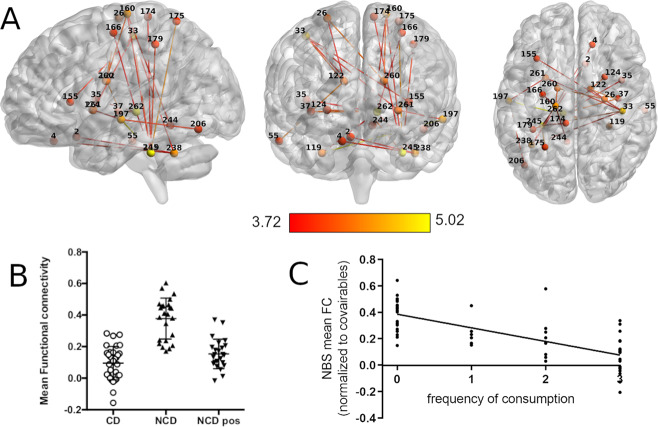
Network with reduced functional connectivity (FC) in habitual coffee drinkers as uncovered by NBS. **A** Sagittal, coronal, and axial view of the network with nodes and edges colored in red–yellow color scheme representing the statistical *t* value of the difference between groups. **B** Scatter plot of the mean FC within the significant network for each experimental group. **C** Associations of frequency of consumption of caffeinated beverages with the mean FC of the network found in NBS.

From the connectomics analysis done using NBS, a single network of significantly higher connectivity was found in the NCD group (pre-coffee) between the thresholds of 0.005 and 0.0005 (for statistics of all thresholds see Supplementary Table 2). For ease of visualization, we present only the results found at the highest significant threshold of *p* = 0.0005 (*t*(threshold) = 3.71, df = 54, *p*(network) = 0.043, Hedge’s *g* = 1.08 (large effect size), 24 nodes, 46 edges; Fig. 2A). The full list of nodes with significant different edges between groups across all thresholds can be found in Supplementary Table 3. Of these we highlight the Thalamus (nodes #262 and #126), the Cerebellum (left anterior Culmen #245 and bilateral Tonsils #238 and #119), the right Postcentral Gyrus (#33), the left Middle Temporal Gyrus (#197), the left Precentral Gyrus (#160), and the bilateral Caudate (#260 and #122) and Putamen (#124 and #261) as having the most strongly affected connections within the network.

When observing the average network connectivity from this network, NCD post-coffee drink displayed a significant reduction in connectivity (Fig. 2B), leading to a profile more similar to CD (*p* = 0.037, *t* = 2.13, df = 54) than to NCD pre-coffee drink (*p* = 1.3 × 10^−7^, *t* = 7.4, df = 23). NBS mean FC was negatively associated with frequency of caffeine consumption (*p* < 0.001; *β* = −0.101; adjusted *R*^2^ = 0.506; Fig. 2C). Detailed statistics can be found in Supplementary Table 1.

### Dynamic FC

**Fig. 3:**
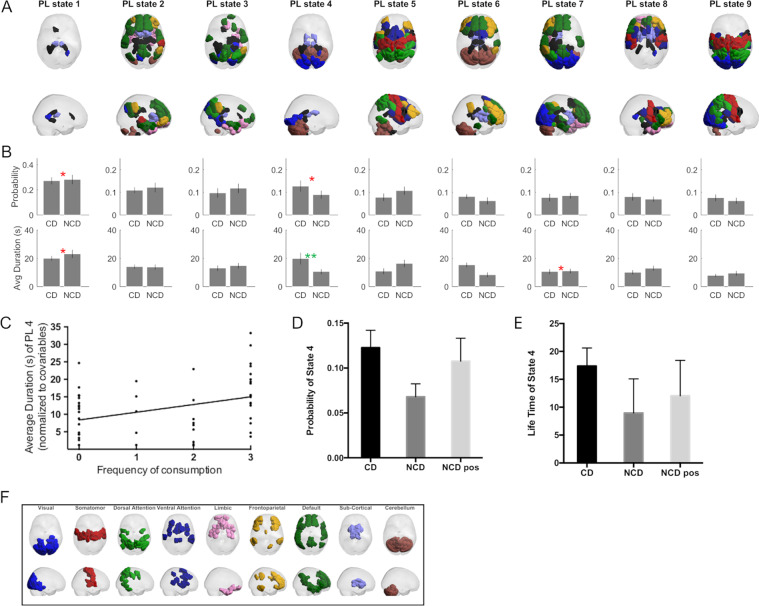
LEiDA results for *k* = 9 of the k-means clustering algorithm and group comparisons of state probability and life time between coffee and non-coffee drinkers. **A** sagittal and axial views representing the state anatomical areas of each phase locked (PL) state. **B** Bar plot representing the group differences between coffee and non-coffee drinkers. Differences of *p* < 0.05 are indicated in red, while multiple comparison surviving effects are indicated in green. **C** Associations of frequency of consumption of caffeinated beverages with the average duration (in seconds) of PL state 4. **D** Bar plot of the probability of state 4 for the CD, NCD, and NCD post caffeine consumption groups. **E** Life time of state 4 for the CD, NCD, and NCD post caffeine consumption groups. **F** Colored labels used to match each anatomical area of the PL states to different resting state networks.

From the dynamic FC analysis, one functional subsystem (Fig. 3A, PL state 4) was found to last significantly longer in CD (Fig. 3B, 17.95 ± 18.32 s) compared to pre-coffee NCD (8.95 ± 6.13 s) surviving correction for multiple comparisons with a corrected *p* = 0.038 and a medium effect size with Hedge’s *g* = 0.62. No BOLD phase-locking state was found to significantly differ in terms of probability of occurrence (see Supplementary Table 4 for all *p* values for all partition models).

This BOLD phase-locking state, corresponding to the fourth most probable state when partitioning the data into nine states, comprises a large number of nodes in the cerebellum, visual network as well as several subcortical nodes such as the bilateral thalamus and parahippocampal gyrus (mapped and color coded through the reference shown in Fig. 3F). While this was the only result that survived correction for multiple comparisons, it is relevant to note that the equivalent LEiDA state for *k* = 10 is just below the threshold (*p* = 0.051, Supplementary Table 4 and Supplementary Figs. 2 and 3). Furthermore, LEiDA lifetime results were positively correlated with frequency of caffeine consumption (*p* = 0.012; *β* = 2.176; adjusted *R*^2^ = 0.083; Fig. 3C).

After drinking coffee, both the lifetime and the probability of this state in NCD became closer to the values observed in CD, with the probability not being significantly different from CD (*p* = 0.5, *t* = 0.67, df = 54), while being significantly higher than NCD pre-coffee (*p* = 0.037, *t* = 2.31, df = 23, Fig. 3D). For the life time of state 4, post-coffee drink NCD were not significantly different from CD (*p* = 0.177, *t* = 1.37, df = 54) nor the pre-drink NCD (*p* = 0.107, *t* = 1.68, df = 23, Fig. 3E). All results across the different *k’*s can be found in Supplementary Figs. 2 and 3 and Supplementary Table 4.

### Effect of habitual caffeine consumption on psychological data

The association between coffee consumption and stress, anxiety, and depression (DASS-21) was assessed. When comparing CD and NCD groups, only stress was significantly different between groups (stress—*p* = 0.025; *Z* = 2.237; *r* = 0.307; anxiety—*p* = 0.851; *Z* = −0.188; *r* = −0.026; depression—*p* = 0.085; *Z* = 1.724; *r* = 0.237), with CD showing higher levels of stress than NCD (median (Med) = 6.0; interquartile range (IQR) = 6.0 vs Med = 4.0; IQR = 4.0, respectively). Of notice, particular items of the DASS-21 Stress subscale that can be related to arousal were increased in CD. Items #1 and #12, which measure difficulty to relax, presented statistically significant differences (*p* = 0.007, Mann–Whitney test), while item #8, that relates to nervous arousal, presented a trend in the same direction (*p* = 0.083). Interestingly, item #7 (Anxiety subscale), that is associated with skeletal musculature, despite not achieving a statistically significant difference between groups, tended to be lower in CD (*p* = 0.113), suggesting a segregation between the motor and arousal loops.

**Fig. 4:**
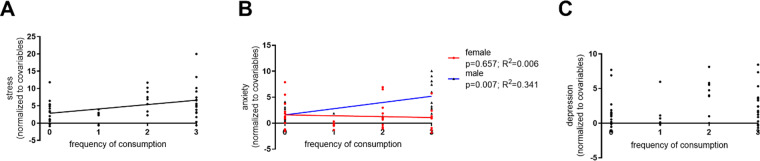
Graphical depiction of the linear regressions associating frequency of consumption of caffeinated products with self-report variables. Associations of frequency of consumption of caffeinated products with the DASS-21 subscales of stress (**A**) and anxiety (**B**), and non-significant association with the depression subscale (**C**).

When assessing the effects of frequency of caffeine consumption in self-reported variables (controlling for sex, age, and education), the positive correlation with stress was maintained (*p* = 0.004; *β* = 1.292; adjusted *R*^2^ = 0.135; Fig. 4A). Moreover, a sex by anxiety interaction was found (*p* = 0.023; *β* = 0.683; adjusted *R*^2^ = 0.085; Fig. 4B), which seems to be driven by a positive correlation in males. No significant effects were found for the depression subscale (*p* = 0.128; *β* = 0.450; adjusted *R*^2^ = 0.108; Fig. 4C). Detailed statistics can be found in Supplementary Table 1.

## Discussion

Herein we describe for the first time the effects of habitual coffee consumption on the human brain networks. We show that habitual CD have different patterns of FC in comparison with NCD. Our rs-fMRI analysis revealed decreased FC of the somatosensory and limbic networks in CD that correlated with the frequency of consumption of caffeinated products. Such changes were replicated in NCD after a single coffee, suggesting possible causality between coffee intake and altered patterns of brain network connectivity. Previous studies have described a reduction of similar RSN connectivity after acute caffeine ingestion [25, 44].

Decreased FC in somatosensory and related networks in CD likely represents a more efficient and beneficial pattern of connections with respect to motor control and alertness; importantly this fits our findings of trends of increased scores in CD in the specific items of the DASS-21 scale that measure these dimensions. The other network impacted by coffee intake was the limbic network, which is involved in processing the sensory input from the external and internal environment which, by modulating memory and motivation, determine emotional, autonomic, motor, and cognitive responses [45]. A previous resting-state PET study showed reduced metabolic activity in components of this network after caffeine ingestion [18] and a study using a hedonic fMRI task showed decreased activation in neuronal areas associated with memory and reward [46] in caffeine consumers compared to non-consumers; the present FC data are consistent with those reports.

Analysis of the global functional connectome using NBS revealed a network impacted by the habitual consumption of caffeine. This widespread network of reduced FC comprised cerebellar, subcortical (striatal and thalamic), and motor cortex regions, partially matching previously reported effects of acute caffeine ingestion [24, 25]. Interestingly, there is a clear bilateral involvement of striatal nodes and of the thalamus which, respectively, have the highest densities of A2A and A1 receptors in the brain [47, 48]. The action of caffeine in these regions has an influence on cortico-striatal-thalamic and cerebellar-thalamocortical loops that are relevant for a variety of neuronal processes. Thus, the observed decrease in FC at rest in this network in regular caffeine-ingesting individuals reveals greater segregation of these areas with less inter-regional dependencies, favoring greater efficiency within these loops. It is relevant to note here that, even though A1 and A2A receptors are thought to mediate differential actions [49], similar effects were observed in both loops. This likely reflects the fact that fMRI provides proxy aggregate measurements of functional connections among a network of brain areas.

A previous study reported that caffeine increases brain entropy, indicating higher information processing capacity across the cerebral cortex [23]. Our LEiDA analysis revealed a dynamic state involving several cerebellar and subcortical areas, with a longer average lifetime in habitual CD. This network comprises several nodes, including the cerebellum, thalamus, and parahipocampal, lingual, and inferior occipital gyri which are relevant in the context of caffeine consumption—caffeine is known to decrease mind wandering [50] and to increase attention, alertness, and arousal [51]. In fact, the nodes implicated in this network are linked by visual processing; among these, the thalamus is critical for distributing cognitive control [52]. The lingual and inferior occipital gyrus are also implicated in visual processing, while the parahippocampus is involved in memory encoding and retrieval [20, 21]; the latter may explain why caffeine reportedly facilitates memory processes [9]. Lastly, evidence of strong rsFC between the cerebellum, known to be also implicated in sensory processing [53] and a number of sensorial cortices [54], explains the observed increased visual alertness/attention and readiness to sensorial perception among CD individuals. While similar findings have been previously reported [6], only one other study assessed habitual CD using MRI, and did not characterize changes in FC [26]. Importantly, similarly to the other neuroimaging findings, a common pattern of connectivity dynamics was found in CD individuals and NCD subjects who drank a single coffee before scanning.

In order to provide a link with other neuropsychologic dimensions, we also assessed our subjects in the DASS-21. Interestingly, we observed habitual CD to display increased levels of stress; there was a clear positive association between the indices of stress and the amount of consumption of caffeinated drinks. Interestingly, items of the DASS-21 sub-score that showed greater variance between CD vs NCD were those related with difficulty to relax (items #1 and #12), and those related to nervous arousal (item #8), consistent with the common attribution of alertness and arousal to coffee intake. It also deserves to be mentioned that, despite the display of a higher anxiety among CD (particularly in males), there was a decrease in DASS-21 item (#7) which matches the effects on the skeletal muscles in CD; this, in turn, fits the findings of better segregation of the above-described loops. The present results extend previous studies that described an association between coffee/caffeine consumption and stress and anxiety [1, 13, 16, 55] and sex [13, 16]. It is important to note, however, that causality cannot be inferred from our study design. Our results are open to two interpretations: higher coffee/caffeine consumption leads to increased stress and anxiety; or, alternatively, higher stress and anxiety induce higher coffee/caffeine consumption. Moreover, given that resting-state studies using stress and anxiety samples have shown both decreases and increases in FC [56–58], the possibility that coffee/caffeine consumption elicits decreases in FC or compensates for FC beyond a certain threshold, must also be considered. While the first possibility is in line with studies showing increased anxiety upon both acute caffeine administration in humans [1, 12] and prolonged ingestion in rodents [59] reports that greater caffeine consumption under periods of stress may help maintain synaptic homeostasis [60] as well as prevent mood disorders warrant further study in future.

The methodologies applied in the present study do not allow us to draw precise relationships between the psychological and neuroimaging results and the dosage and metabolism of caffeine among individual subjects. To study the individual responses to the acute and chronic effects of caffeinated product intake would be a complex undertaking, requiring subjects to adapt their daily habits and strict abstinence regimens. Based on our experience, recruitment of subjects for a properly balanced study is also difficult since NCD subjects are insufficiently motivated to engage in studies on the actions of caffeine. Nevertheless, we are currently developing alternative strategies that would allow us to deliver calibrated doses of caffeine during fMRI scanning sessions to better discriminate its effects from other factors (e.g., stress). Our future work will also examine inter-individual differences in response to caffeine consumption, the subjective experience of coffee consumption, as well as the influence of additional factors as the consumption of alcohol and tobacco. Despite such gaps, the data presented here represent a contribution to the knowledge of the “caffeinated brain” and how these changes underlie the behavioral effects triggered by coffee intake, with implications for physiological and pathological conditions.

## Supplementary information

Supplemental Materials

## Data Availability

In-house scripts used in the NBS analysis are fully available online at open science framework website (https://osf.io/qepc8/) and LEiDA scripts at github (https://github.com/juanitacabral/LEiDA).
