# Supplementary material for: Habitual coffee drinkers display a distinct pattern of brain functional connectivity
Source: Mol Psychiatry. 2021 Apr 20;26(11):6589–98. doi: 10.1038/s41380-021-01075-4 (PMC8760045; doi:10.1038/s41380-021-01075-4)
Supplement: Supplementary file 1 — Supplemental Materials [file 41380_2021_1075_MOESM1_ESM.pdf]

Supplementary methods

Anatomical data preprocessing

The T1w image was corrected for intensity non-uniformity (INU) with N4BiasFieldCorrection (1), distributed with ANTs 2.2.0 ((2), RRID:SCR_004757), and used as T1w-reference throughout the workflow. The T1w-reference was then skull-stripped with a Nipype implementation of the *antsBrainExtraction.sh* workflow (from ANTs), using OASIS30ANTs as target template. Brain tissue segmentation of cerebrospinal fluid (CSF), white-matter (WM) and gray-matter (GM) was performed on the brain-extracted T1w using *fast* (FSL 5.0.9, RRID:SCR_002823, (3)). Brain surfaces were reconstructed using *recon-all* (FreeSurfer 6.0.1, RRID:SCR_001847, (4)), and the brain mask estimated previously was refined with a custom variation of the method to reconcile ANTs-derived and FreeSurfer-derived segmentations of the cortical gray-matter of Mindboggle (RRID:SCR_002438, (5)). Volume-based spatial normalization to standard space (ICBM 152 Nonlinear Asymmetrical template version 2009c [(6), RRID:SCR_008796; TemplateFlow ID: MNI152NLin2009cAsym]) was performed through nonlinear registration with *antsRegistration* (ANTs 2.2.0), using brain-extracted versions of both T1w reference and the T1w template.

Resting-state data preprocessing

For each subject, the following preprocessing steps were performed: First, a reference volume and its skull-stripped version were generated using a custom methodology of fMRIPrep. A deformation field to correct for susceptibility distortions was estimated based on a field map that was co-registered to the BOLD reference, using a custom workflow of fMRIPrep derived from D. Greve's *epidewarp.fsl* script and further improvements of HCP Pipelines (7). Based on the estimated susceptibility distortion, an unwarped BOLD reference was calculated for a more accurate co-registration with the anatomical reference. The BOLD reference was then co-registered to the T1w reference using *bbregister* (FreeSurfer) which implements boundary-based registration (8). Co-registration was configured with nine degrees of freedom to account for distortions remaining in the BOLD reference. Head-motion parameters with respect to the BOLD reference (transformation matrices, and six corresponding rotation and translation parameters) were estimated before any spatiotemporal filtering using *mcflirt* (FSL 5.0.9, (9)). BOLD runs were slice-time corrected using *3dTshift* from AFNI 20160207 ((10), RRID:SCR_005927). The BOLD time-series (slice-time corrected) were resampled onto their original, native space by applying a single, composite transform to correct for head-motion and susceptibility distortions, and finally resampled into *MNI152NLin2009cAsym* space. Several confounding time-series were calculated based on the preprocessed BOLD: framewise displacement (FD), DVARS (rate of change of BOLD signal across the entire brain at each frame of data), and three region-wise global signals. FD and DVARS are calculated for each functional run, both using their implementations in Nipype (following the definitions by (11)). The three global signals were extracted within the CSF, the WM, and the whole-brain masks. Additionally, a set of physiological regressors were extracted to allow for component-based noise correction (CompCor, (12)). Principal components were estimated after high-pass filtering the preprocessed BOLD time-series (using a discrete cosine filter with 128s cut-off) for the anatomical variant (aCompCor). A mask covering the subcortical regions was obtained by heavily eroding the brain mask, which ensures it does not include cortical GM regions. For aCompCor, components were calculated

within the intersection of the aforementioned mask and the union of CSF and WM masks calculated in T1w space, after their projection to the native space of each functional run (using the inverse BOLD-to-T1w transformation). Components were calculated separately within the WM and CSF masks. For each CompCor decomposition, the k components with the largest singular values were retained, such that the retained components' time series were sufficient to explain 50 percent of variance across the nuisance mask (CSF, WM, combined, or temporal). The remaining components were dropped from consideration. The mean CSF and WM signals, as well as the first 6 aCompCor components, the FD and the DVARS were regressed as confounds from the BOLD data using *fslregfilt*. Movement was considered excessive with a mean FD > 0.25, but none of the subjects exceeded this threshold and thus no subjects had to be excluded because of this. Finally, *fslmaths* was used to spatially smooth (with a FWHM kernel of 6mm) and band-pass filter (between 0.01 and 0.08 Hz) the resulting time-series.

Independent Component Analysis

Probabilistic ICA is a fully data-driven approach that enables the isolation of components based on the temporal correlation of the corresponding areas, while maximizing the spatial independence between components. Then, dual-regression analysis was performed to estimate the subject-specific components that correspond to the group-wise RSNs. Because the probabilistic ICA approach may identify noisy components corresponding to non-biological signal, such as movement artifacts, the independent components were selected after visual inspection of their spatial distribution (13). Specifically, components that were mainly present in regions that do not generate the BOLD signal (white matter, ventricles or outside the brain) were excluded from the analysis.

Static functional connectivity analysis

This approach provides a correction equivalent to the FWE-R by estimating the probability of identifying, in a random permutation of the tested data, networks with a larger extent than the ones identified in the hypothesis tested and is done in two phases. The first step tests the statistical hypothesis at each point of the matrix, which is then filtered by a user-determined statistical edge threshold. Significant network components are identified as sets of threshold-surviving connections between nodes, such that each node in the network can be reached from any other, through significant connections. The component size is calculated as the number of significant connections. While the connection threshold is not directly determinant of the network significance, it determines the possible extent of the network, with lower significance edge thresholds revealing larger and more widespread networks and higher thresholds resulting in smaller and more focused effects. As such, following the toolbox authors' recommendation we explored edge thresholds between 0.005 and 0.0001, with two intermediate steps of 0.001 and 0.0005. Then, in step 2 random permutations of the data-set are created and the same methodology as in step 1 is applied to each permutation, determining the size of network components found. Finally, the calculated distribution of components size is used to estimate the probability of finding random components with a size greater than the one found in our hypothesis.

Dynamic functional analysis

The iFC was calculated as the phase coherence using the Hilbert transform, generating for each subject a 3D FC matrix. To study temporal patterns, we used the leading Eigenvector of each time point of the connectivity matrix, capturing the dominant

pattern of FC for each timepoint. Using this Eigenvector, we studied the evolution of the functional dynamics of these states. FC patterns, as represented by the leading Eigenvector, are typically expected to be recurrent, repeating themselves over time. In the current work we are interested in understanding if the habitual consumption of caffeine affects the probability of a subject entering each brain state, as well as the time that such a state is dominant. For this purpose, we applied a k-means clustering algorithm upon the entire dataset, clustering the data into clusters representing recurrent phase locked (PL) FC states. Applying such an algorithm typically requires the user to choose a number of clusters (k). As it is not possible for us to empirically determine this parameter, we explored a range of possibilities running the algorithm for values of k between 3 and 15 thus covering a wide range of possible states. For each state of each iteration, the probability was calculated as the number of times each state was dominant and the lifetime (LT) as the average duration during which a state was dominant, in seconds. Statistical testing of both parameters was done through permutation testing, comparing the two groups using a two-sample t-test, generating 5000 permutations of the groups and calculating the distribution of differences. Results were considered significant at $p<0.05$, with multiple comparison correction for the number of clusters tested at each k (for the iteration where $k=10$, we corrected for those 10 comparisons).

References

1. Tustison NJ, Avants BB, Cook PA, Yuanjie Zheng, Egan A, Yushkevich PA, et al. N4ITK: Improved N3 Bias Correction. IEEE Trans Med Imaging. 2010 Jun;29(6):1310–20.

2. Avants B, Epstein C, Grossman M, Gee J. Symmetric diffeomorphic image registration with cross-correlation: Evaluating automated labeling of elderly and neurodegenerative brain. Medical Image Analysis. 2008 Feb;12(1):26–41.

3. Zhang Y, Brady M, Smith S. Segmentation of brain MR images through a hidden Markov random field model and the expectation-maximization algorithm. IEEE Trans Med Imaging. 2001 Jan;20(1):45–57.

4. Dale AM, Fischl B, Sereno MI. Cortical Surface-Based Analysis. NeuroImage. 1999 Feb;9(2):179–94.

5. Klein A, Ghosh SS, Bao FS, Giard J, Häme Y, Stavsky E, et al. Mindboggling morphometry of human brains. Schneidman D, editor. PLoS Comput Biol. 2017 Feb 23;13(2):e1005350.

6. Fonov V, Evans A, McKinstry R, Almli C, Collins D. Unbiased nonlinear average age-appropriate brain templates from birth to adulthood. NeuroImage. 2009 Jul;47:S102.

7. Glasser MF, Sotiropoulos SN, Wilson JA, Coalson TS, Fischl B, Andersson JL, et al. The minimal preprocessing pipelines for the Human Connectome Project. NeuroImage. 2013 Oct;80:105–24.

8. Greve DN, Fischl B. Accurate and robust brain image alignment using boundary-based registration. NeuroImage. 2009 Oct;48(1):63–72.

9. Jenkinson M, Bannister P, Brady M, Smith S. Improved Optimization for the Robust and Accurate Linear Registration and Motion Correction of Brain Images. NeuroImage. 2002 Oct;17(2):825–41.

10. Cox RW. AFNI: Software for Analysis and Visualization of Functional Magnetic Resonance Neuroimages. Computers and Biomedical Research. 1996 Jun;29(3):162–73.

11. Power JD, Mitra A, Laumann TO, Snyder AZ, Schlaggar BL, Petersen SE. Methods to detect, characterize, and remove motion artifact in resting state fMRI. NeuroImage. 2014 Jan;84:320–41.

12. Behzadi Y, Restom K, Liau J, Liu TT. A component based noise correction method (CompCor) for BOLD and perfusion based fMRI. NeuroImage. 2007 Aug;37(1):90–101.

13. Horowitz-Kraus T, DiFrancesco M, Kay B, Wang Y, Holland SK. Increased resting-state functional connectivity of visual- and cognitive-control brain networks after training in children with reading difficulties. NeuroImage: Clinical. 2015;8:619–30.

Supplementary Data

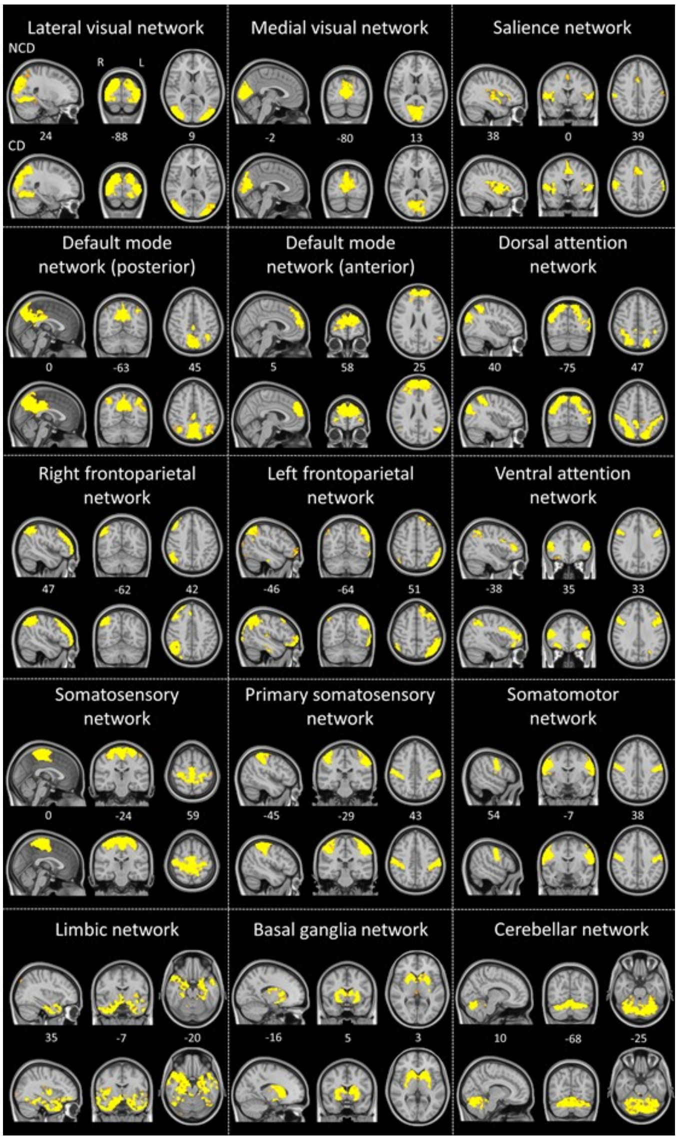

Supplementary Fig. 1. Resting-state networks identified through probabilistic ICA. Within each RSN, the NCD group is always shown at the top, and the CD group at the bottom. Coordinates are x, y, z MNI coordinates and apply for the slices shown for both groups (top and bottom). A general trend for lower FC patterns can be observed in the CD group.

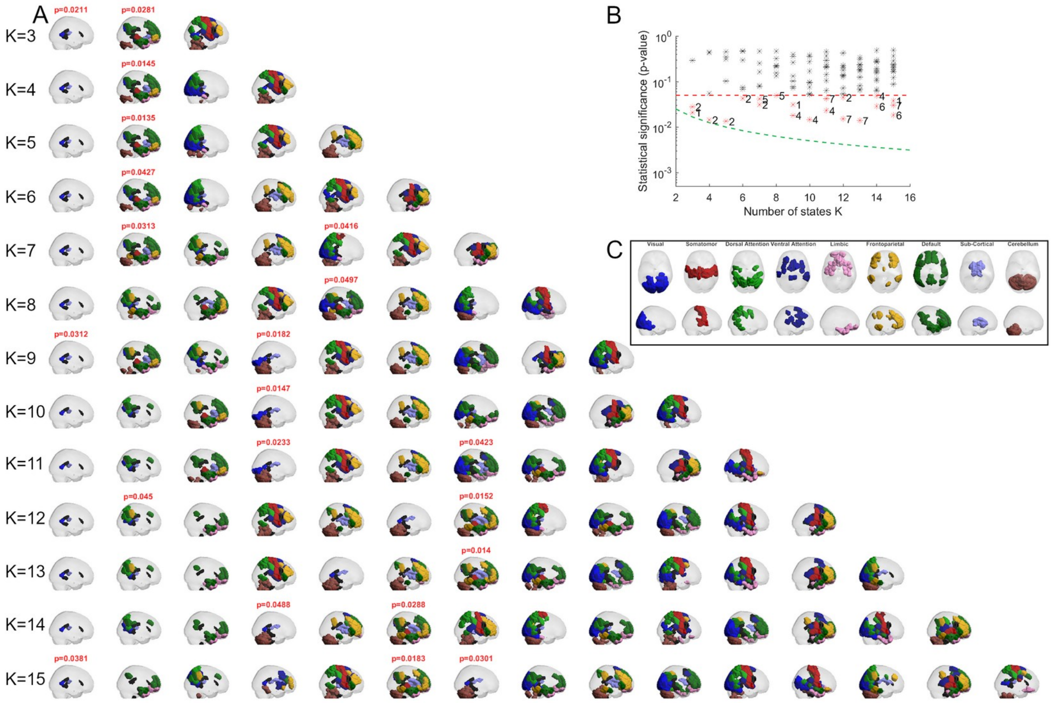

Supplementary Fig. 2. Phase Lock states and significance of the comparison between CD and NCD for state probability for all K's between 3 and 15. A – Saggital view over a mesh of the areas composing each state, color coded according to their overlap with different RSNs and matching significance for the comparison of state probability with results surviving a threshold of $p < 0.05$ in red; **B** – Plots of the p-value for each state of all threshold. Threshold of $p < 0.05$ indicated in red and multiple comparison correction for the number of states is indicated in green. **C** – Color code map matching each anatomical area of each state to the different resting state networks.

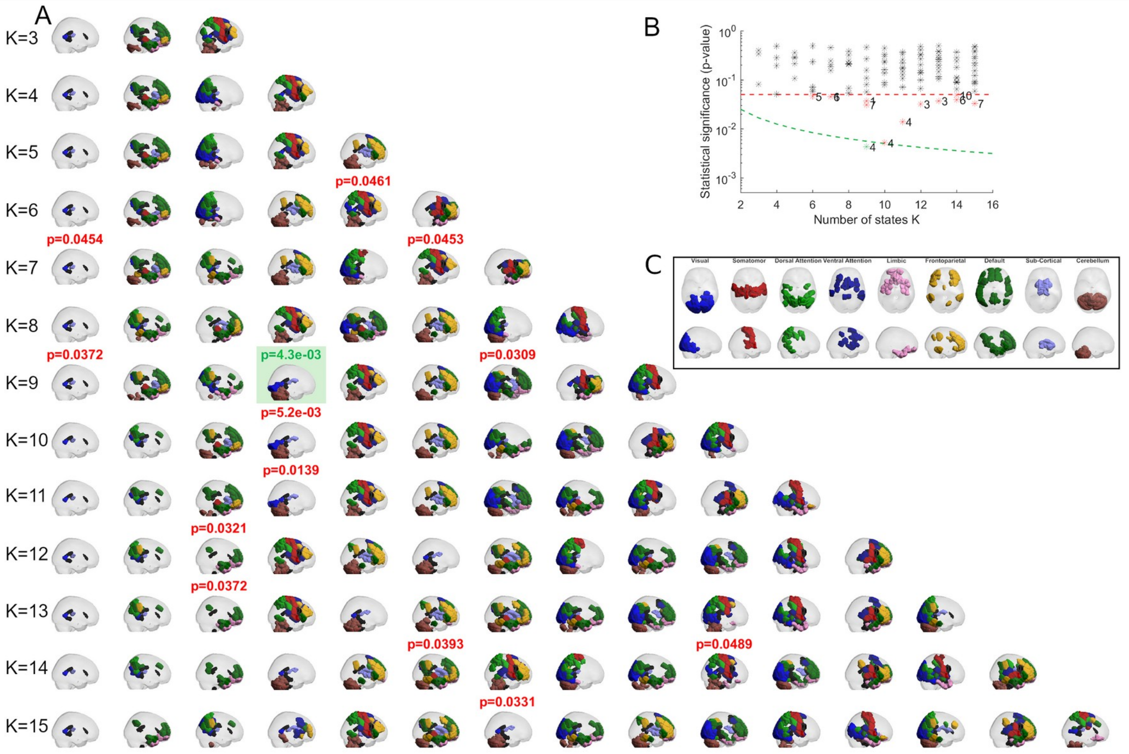

Supplementary Fig. 3. Phase Lock states and significance of the comparison between CD and NCD for state lifetime for all K's between 3 and 15. A – Saggital view over a mesh of the areas composing

each state, color coded according to their overlap with different RSNs and matching significance for the comparison of state lifetime with results surviving a threshold of $p < 0.05$ in red; **B** – Plots of the p-value for each state of all threshold. Threshold of $p < 0.05$ indicated in red and multiple comparison correction for the number of states is indicated in green. **C** – Color code map matching each anatomical area of each state to the different resting state networks.

Supplementary Table 1. Statistical data regarding the associations between frequency of caffeine consumption and psychological and neuroimaging data. Results of multiple linear regressions in which the independent variable of interest is frequency of consumption (freq) and the dependent variable are self-report subscales, functional connectivity (FC) on ICA or NBS, lifetime (LT) on LEiDA, or the volume of regions-of-interest (ROI) are shown. All regressions are controlled for sex, age and education, and the sex-freq interaction was also explored. Freq - frequency of consumption of caffeinated beverages; EADS-21 - depression, anxiety and stress scale; ICA - independent-component analysis; NBS - network based statistics; FC - functional connectivity; LT - lifetime.

		EADS-21			ICA FC		NBS FC	LEiDA
		stress	depression	anxiety	right precuneus	right insula	mean FC	LT
p	intercept	0.001	0.015	0.007	0.016	<0.001	<0.001	<0.001
	freq	0.004	0.128	0.078	0.003	<0.001	<0.001	0.012
	sex	0.187	0.637	0.102	0.161	0.053	0.129	0.956
	age	0.173	0.006	0.491	0.539	0.166	0.971	0.200
	education	0.203	0.386	0.112	0.921	0.832	0.802	0.430
	sex x freq	0.171	0.507	0.023	0.457	0.153	0.055	0.695
β	intercept	3.098	1.445	1.589	2.282	7.159	0.377	8.479
	freq	1.292	0.450	0.514	-1.433	-2.384	-0.101	2.176
	sex	-1.127	-0.273	-0.954	-1.315	2.286	0.043	-0.095
	age	-0.075	-0.105	-0.025	-0.036	-0.105	<0.001	0.141
	education	-0.231	0.108	-0.199	0.019	-0.052	-0.001	-0.287

	sex x freq	0.599	0.198	0.683	0.348	-0.851	-0.028	0.337
R^2		0,220	0.193	0.172	0.240	0.336	0.552	0.171
adjusted R^2		0,135	0.108	0.085	0.162	0.267	0.506	0.083

Supplementary Table 2. Results of the functional connectomics analysis using NBS. Here we report for each of the four thresholds used the p-value of the significant networks, the Hedge's g as a measure of effect size, the number of nodes, the number of edges in the network and the mean network FC for each group. N.S-non significant.

Threshold (t,p)	p (network)	g	N Nodes	N edges	FC CD	FC NCD
2.92, 0.005	0.024	0.89	174	352	0.12	0.36
3.48, 0.001	0.032	1.03	58	64	0.08	0.35
3.71, 0.0005	0.043	1.09	24	23	0.09	0.38
4.21, 0.0001	N.S	-	-	-	-	-

Supplementary Table 3. Nodes of the network, built from the Shen atlas, with connecting edges that have a significant group effect between CD and NCD. Results are represented for the three thresholds with significant results. Here we represent the name and number of each node as well as the sum t-statistic value (t) over it's significant connections.

#Node	Name			*p*=0.005	0.001	0.0005
1	Right	Cerebrum	Superior_Frontal_Gyrus	12.3	-	-
2	Right	Cerebrum	Rectal_Gyrus	12.9	3.8	3.8
3	Right	Cerebrum	Medial_Frontal_Gyrus	7.1	3.7	-
4	Right	Cerebrum	Inferior_Frontal_Gyrus	6.9	3.9	3.9
5	Right	Cerebrum	Anterior_Cingulate	3.8	-	-
6	Right	Cerebrum	Medial_Frontal_Gyrus	9.0	-	-
7	Right	Cerebrum	Superior_Frontal_Gyrus	9.5	-	-
8	Right	Cerebrum	Middle_Frontal_Gyrus	3.1	-	-
9	Right	Cerebrum	Middle_Frontal_Gyrus	6.0	-	-
11	Right	Cerebrum	Superior_Frontal_Gyrus	19.6	-	-
16	Right	Cerebrum	Inferior_Frontal_Gyrus	6.2	-	-
17	Right	Cerebrum	Middle_Frontal_Gyrus	10.2	7.3	-
18	Right	Cerebrum	Inferior_Frontal_Gyrus	20.4	7.8	-
19	Right	Cerebrum	Middle_Frontal_Gyrus	6.7	-	-
21	Right	Cerebrum	Inferior_Frontal_Gyrus	3.1	-	-
22	Right	Cerebrum	Middle_Frontal_Gyrus	3.2	-	-
24	Right	Cerebrum	Medial_Frontal_Gyrus	12.6	-	-
26	Right	Cerebrum	Precentral_Gyrus	17.6	8.0	8.0
27	Right	Cerebrum	Precentral_Gyrus	19.0	-	-
29	Right	Cerebrum	Superior_Frontal_Gyrus	15.6	-	-
32	Right	Cerebrum	Precentral_Gyrus	12.4	-	-
33	Right	Cerebrum	Postcentral_Gyrus	44.8	15.6	15.6
34	Right	Cerebrum	Extra-Nuclear_Sub_Lobar	13.5	7.2	-
35	Right	Cerebrum	Insula	3.7	3.7	3.7
37	Right	Cerebrum	Claustrum	4.2	4.2	4.2
38	Right	Cerebrum	Inferior_Parietal_Lobule	3.1	-	-
39	Right	Cerebrum	Postcentral_Gyrus	3.4	-	-
41	Right	Cerebrum	Postcentral_Gyrus	3.2	-	-
43	Right	Cerebrum	Superior_Parietal_Lobule	3.2	-	-
45	Right	Cerebrum	Postcentral_Gyrus	2.9	-	-
47	Right	Cerebrum	Inferior_Parietal_Lobule	3.3	-	-
52	Right	Cerebrum	Superior_Temporal_Gyrus	9.6	-	-
54	Right	Cerebrum	Middle_Temporal_Gyrus	17.8	11.1	-
55	Right	Cerebrum	Inferior_Temporal_Gyrus	7.4	4.4	4.4
56	Right	Cerebrum	Inferior_Temporal_Gyrus	3.1	-	-
57	Right	Cerebrum	Middle_Temporal_Gyrus	7.0	3.8	-
58	Right	Cerebrum	Inferior_Temporal_Gyrus	3.0	-	-

61	Right	Cerebrum	Superior_Temporal_Gyrus	28.4	-	-
62	Right	Cerebrum	Gray_Matter_Brodmann	15.6	-	-
63	Right	Cerebrum	Superior_Temporal_Gyrus	30.5	14.8	-
64	Right	Cerebrum	Inferior_Temporal_Gyrus	3.2	-	-
66	Right	Cerebrum	Fusiform_Gyrus	8.9	-	-
67	Right	Cerebellum	Declive	3.0	-	-
69	Right	Cerebrum	Inferior_Temporal_Gyrus	3.0	-	-
71	Right	Cerebellum	Culmen	9.5	-	-
72	Right	Cerebellum	Declive	28.9	3.7	-
73	Right	Cerebrum	Middle_Occipital_Gyrus	6.7	3.5	-
74	Right	Cerebrum	Inferior_Occipital_Gyrus	12.4	-	-
75	Right	Cerebrum	PreCuneus	3.3	-	-
76	Right	Cerebrum	Lingual_GyrusGray	6.0	-	-
77	Right	Cerebrum	CuneusGray	3.5	-	-
78	Right	Cerebrum	Middle_Occipital_Gyrus	27.5	-	-
79	Right	Cerebrum	Lingual_Gyrus	7.0	-	-
80	Right	Cerebrum	Cuneus	22.1	-	-
82	Right	Cerebrum	Cuneus	6.5	-	-
92	Right	Cerebrum	Uncus	3.0	-	-
96	Right	Cerebrum	Parahippocampal_Gyrus	3.1	-	-
97	Right	Cerebrum	Uncus	6.7	3.8	-
101	Right	Cerebellum	Culmen	22.7	-	-
102	Right	Cerebellum	Tuber	9.9	-	-
103	Right	Cerebellum	I-IV	3.5	3.5	-
104	Right	Cerebellum	Cerebellar_Tonsil	3.7	3.7	-
105	Right	Cerebellum	Inferior_Semi-Lunar_Lobule	6.2	-	-
106	Right	Cerebellum	Declive	41.9	7.5	-
110	Right	Cerebellum	V-VI	19.5	7.3	-
111	Right	Cerebellum	Pyramis	5.9	-	-
113	Right	Cerebellum	Cerebellar_Tonsil	29.0	-	-
114	Right	Cerebellum	Pyramis	6.2	-	-
117	Right	Cerebellum	Cerebellar_Tonsil	21.7	-	-
119	Right	Cerebellum	Cerebellar_Tonsil	8.7	8.7	8.7
121	Right	Cerebrum	Caudate_Body	6.2	-	-
122	Right	Cerebrum	Caudate_Body	17.8	8.1	8.1
124	Right	Cerebrum	Nucleus_Putamen	22.7	4.1	4.1
125	Right	Cerebrum	Nucleus_Putamen	3.0	-	-
126	Right	Cerebrum	Thalamus	32.8	3.9	-
128	Right	Cerebrum	Thalamus	3.0	-	-
133	Right	Cerebellum	Cerebellar_Tonsil	3.4	-	-
134	Left	Cerebrum	Anterior_Cingulate	3.1	-	-
137	Left	Cerebrum	Rectal_Gyrus	3.2	-	-
138	Left	Cerebrum	Medial_Frontal_Gyrus	13.2	-	-
139	Left	Cerebrum	Superior_Frontal_Gyrus	3.1	-	-
140	Left	Cerebrum	Medial_Frontal_Gyrus	3.0	-	-
141	Left	Cerebrum	Medial_Frontal_Gyrus	6.9	3.6	-
142	Left	Cerebrum	Middle_Frontal_Gyrus	15.6	-	-
143	Left	Cerebrum	Middle_Frontal_Gyrus	15.5	-	-
144	Left	Cerebrum	Middle_Frontal_Gyrus	3.0	-	-
146	Left	Cerebrum	Middle_Frontal_Gyrus	28.9	-	-
147	Left	Cerebrum	Middle_Frontal_Gyrus	6.1	-	-
148	Left	Cerebrum	Superior_Frontal_Gyrus	12.1	-	-
149	Left	Cerebrum	Superior_Frontal_Gyrus	6.4	-	-
150	Left	Cerebrum	Medial_Frontal_Gyrus	5.9	-	-
151	Left	Cerebrum	Inferior_Frontal_Gyrus	27.8	15.4	-
152	Left	Cerebrum	Middle_Frontal_Gyrus	12.7	-	-
153	Left	Cerebrum	Inferior_Frontal_Gyrus	3.3	-	-
154	Left	Cerebrum	Middle_Frontal_Gyrus	9.0	-	-
155	Left	Cerebrum	Insula	7.0	3.9	3.9
157	Left	Cerebrum	Inferior_Frontal_Gyrus	3.5	3.5	-
158	Left	Cerebrum	Precentral_Gyrus	15.6	-	-
160	Left	Cerebrum	Precentral_Gyrus	24.5	15.4	11.8
161	Left	Cerebrum	Cingulate_Gyrus	6.5	3.6	-
162	Left	Cerebrum	Superior_Frontal_Gyrus	10.3	-	-
163	Left	Cerebrum	Precentral_Gyrus	6.1	-	-
166	Left	Cerebrum	Middle_Frontal_Gyrus	13.9	10.9	3.7
167	Left	Cerebrum	Precentral_Gyrus	20.3	7.2	-
169	Left	Cerebrum	Insula	6.4	-	-
170	Left	Cerebrum	Claustrum	3.3	-	-
171	Left	Cerebrum	Postcentral_Gyrus	3.7	3.7	-
172	Left	Cerebrum	Postcentral_Gyrus	9.5	-	-
173	Left	Cerebrum	Insula	6.7	-	-
174	Left	Cerebrum	Paracentral_Lobule	7.1	3.8	3.8
175	Left	Cerebrum	Superior_Parietal_Lobule	7.5	4.5	4.5
179	Left	Cerebrum	Inferior_Parietal_Lobule	3.9	3.9	3.9
180	Left	Cerebrum	Superior_Temporal_Gyrus	6.1	-	-

182	Left	Cerebrum	Inferior_Parietal_Lobule	6.2	-	-
183	Left	Cerebrum	Superior_Temporal_Gyrus	3.3	-	-
184	Left	Cerebrum	Inferior_Parietal_Lobule	9.2	-	-
185	Left	Cerebrum	Middle_Temporal_Gyrus	3.5	-	-
187	Left	Cerebrum	Middle_Temporal_Gyrus	19.3	4.0	-
188	Left	Cerebrum	Middle_Temporal_Gyrus	3.3	-	-
190	Left	Cerebrum	Fusiform_Gyrus	9.5	3.5	-
191	Left	Cerebrum	Superior_Temporal_Gyrus	26.4	7.6	-
192	Left	Cerebrum	Middle_Temporal_Gyrus	3.4	-	-
193	Left	Cerebrum	Inferior_Temporal_Gyrus	-1.0	-	-
194	Left	Cerebrum	Inferior_Temporal_Gyrus	16.2	3.6	-
195	Left	Cerebrum	Uncus	3.0	-	-
196	Left	Cerebrum	Inferior_Temporal_Gyrus	3.1	-	-
197	Left	Cerebrum	Middle_Temporal_Gyrus	73.7	23.4	12.8
198	Left	Cerebellum	Culmen	3.0	-	-
199	Left	Cerebrum	Inferior_Temporal_Gyrus	16.6	-	-
200	Left	Cerebrum	Fusiform_Gyrus	15.9	3.5	-
201	Left	Cerebellum	Culmen	6.4	-	-
202	Left	Cerebrum	Uncus	3.4	-	-
204	Left	Cerebrum	Middle_Occipital_Gyrus	43.1	8.1	-
205	Left	Cerebrum	Lingual_Gyrus	3.0	-	-
206	Left	Cerebrum	Fusiform_Gyrus	44.0	7.5	3.9
207	Left	Cerebellum	Declive	12.3	-	-
208	Left	Cerebrum	Cuneus	13.1	-	-
209	Left	Cerebrum	Inferior_Temporal_Gyrus	3.3	-	-
210	Left	Cerebrum	Inferior_Occipital_Gyrus	9.3	-	-
211	Left	Cerebrum	Lingual_Gyrus	3.2	-	-
212	Left	Cerebrum	Cuneus	52.5	14.9	-
213	Left	Cerebrum	Lingual_Gyrus	21.5	-	-
214	Left	Cerebrum	Inferior_Occipital_Gyrus	9.5	-	-
215	Left	Cerebrum	Cuneus	3.0	-	-
216	Left	Cerebrum	Posterior_Cingulate	3.1	-	-
220	Left	Cerebrum	Cingulate_Gyrus	9.3	-	-
224	Left	Cerebrum	Cingulate_Gyrus	9.2	-	-
225	Left	Cerebrum	PreCuneus	3.0	-	-
228	Left	Cerebrum	Parahippocampal_Gyrus	3.0	-	-
231	Left	Cerebrum	Parahippocampal_Gyrus	17.4	11.2	-
232	Left	Cerebrum	Parahippocampal_Gyrus	16.2	-	-
233	Left	Cerebrum	Parahippocampal_Gyrus	18.6	-	-
234	Left	Cerebrum	Parahippocampal_Gyrus	9.1	-	-
235	Left	Cerebrum	Uncus	9.9	3.5	-
236	Left	Cerebellum	Inferior_Semi-Lunar_Lobule	15.4	-	-
238	Left	Cerebellum	Cerebellar_Tonsil	73.2	29.5	11.5
241	Left	Cerebellum	Tuber	6.2	-	-
244	Left	Cerebellum	Culmen	6.9	3.8	3.8
245	Left	Cerebellum	Culmen	85.1	38.2	20.2
248	Left	Cerebellum	Declive	24.4	11.9	-
251	Left	Cerebellum	Brainstem	24.9	-	-
252	Left	Cerebellum	Cerebellar_Tonsil	18.6	-	-
254	Left	Cerebellum	V-VI	61.6	10.6	-
255	Left	Cerebellum	I-IV	12.3	-	-
256	Left	Cerebellum	Cerebellar_Tonsil	-2.0	7.3	-
258	Left	Cerebrum	Caudate	24.4	-	-
260	Left	Cerebrum	Caudate	37.8	8.4	8.4
261	Left	Cerebrum	Lentiform_Nucleus_Putamen	42.2	7.6	7.6
262	Left	Cerebrum	Thalamus	46.0	26.7	19.5
265	Left	Brainstem	Brainstem_Midbrain	9.6	-	-
267	Left	Brainstem	Brainstem_Midbrain	28.2	-	-
268	Left	Brainstem	Brainstem_Midbrain	9.6	-	-

Supplementary Table 4. Uncorrected statistical significance at all cluster levels between k = 3 and k = 15 for probability and life time of each state for the comparison between coffee drinkers and non-coffee drinkers. For each clustering level (k in each line) and state (column) we indicate the uncorrected statistical significance. The result that survives correction for multiple comparisons is in bold.

pLT/pP	S 1	S 2	S 3	S 4	S 5	S 6	S 7	S 8	S 9	S 10	S 11	S 12	S 13	S 14	S 15
k=3	0.400/ 0.021	0.081/ 0.028	0.339/ 0.294	-	-	-	-	-	-	-	-	-	-	-	-
k=	0.484/	0.051/	0.195/	0.281/	-	-	-	-	-	-	-	-	-	-	-

4	0.055	0.015	0.447	0.431											
k=5	0.277/0.103	0.106/0.013	0.293/0.456	0.212/0.312	0.360/0.349	-	-	-	-	-	-	-	-	-	-
k=6	0.057/0.075	0.234/0.043	0.062/0.470	0.071/0.297	0.046/0.465	0.493/0.068	-	-	-	-	-	-	-	-	-
k=7	0.045/0.082	0.188/0.031	0.155/0.164	0.205/0.254	0.243/0.042	0.045/0.466	0.454/0.078	-	-	-	-	-	-	-	-
k=8	0.242/0.134	0.208/0.155	0.209/0.227	0.054/0.464	0.067/0.050	0.213/0.322	0.322/0.210	0.384/0.221	-	-	-	-	-	-	-
k=9	0.037/0.031	0.267/0.133	0.108/0.078	**0.004**/0.018	0.079/0.398	0.157/0.265	0.031/0.102	0.458/0.072	0.056/0.359	-	-	-	-	-	-
k=10	0.329/0.095	0.241/0.054	0.303/0.171	0.005/0.015	0.088/0.383	0.228/0.274	0.161/0.075	0.138/0.05	0.442/0.098	0.078/0.357	-	-	-	-	-
k=11	0.366/0.106	0.170/0.064	0.257/0.214	0.014/0.023	0.071/0.366	0.336/0.292	0.127/0.042	0.107/0.143	0.149/0.452	0.180/0.204	0.220/0.235	-	-	-	-
k=12	0.320/0.128	0.138/0.045	0.032/0.227	0.099/0.427	0.409/0.142	0.067/0.072	0.203/0.015	0.480/0.180	0.155/0.218	0.141/0.053	0.268/0.202	0.331/0.135	-	-	-
k=13	0.316/0.125	0.281/0.081	0.037/0.180	0.236/0.340	0.105/0.068	0.492/0.207	0.175/0.014	0.146/0.186	0.116/0.065	0.265/0.308	0.200/0.238	0.379/0.169	0.383/0.234	-	-
k=14	0.180/0.106	0.111/0.063	0.093/0.270	0.107/0.049	0.370/0.274	0.039/0.029	0.284/0.296	0.239/0.166	0.088/0.179	0.049/0.114	0.089/0.067	0.242/0.155	0.180/0.487	0.064/0.194	-
k=15	0.273/0.038	0.153/0.170	0.197/0.167	0.083/0.123	0.372/0.369	0.059/0.018	0.033/0.030	0.089/0.185	0.465/0.284	0.222/0.088	0.093/0.491	0.122/0.240	0.297/0.227	0.481/0.211	0.392/0.200
